# Hunters and Gatherers of Pictures: Why Photography Has Become a Human Universal

**DOI:** 10.3389/fpsyg.2021.654474

**Published:** 2021-06-08

**Authors:** Leopold Kislinger, Kurt Kotrschal

**Affiliations:** ^1^Independent Researcher, Leonding, Austria; ^2^Department of Behavioral Biology and Konrad Lorenz Forschungsstelle, University of Vienna, Vienna, Austria; ^3^Domestication Lab at the Konrad-Lorenz Institute of Ethology, Wolf Science Center, University of Veterinary Medicine, Ernstbrunn, Austria

**Keywords:** photography, psychology, biology, evolution, coping, wellbeing, human universals, social cognition and interaction

## Abstract

Photography is ubiquitous worldwide. We analyzed why people take, share, and use personal photographs, independent of their specific cultural background. These behaviors are still poorly understood. Experimental research on them is scarce. Smartphone technology and social media have pushed the success of photography, but cannot explain it, as not all smartphone features are widely used just because they are available. We analyzed properties of human nature that have made taking and using photographs functional behaviors. We did this based on the four levels, which Nikolaas Tinbergen suggested for analyzing why animals behave in a particular way. Including findings from multiple disciplines, we developed a novel conceptual framework—the “Mental Utilization Hypothesis of Photography.” It suggests that people adopt photography because it matches with core human mental mechanisms mainly from the social domain, and people use photography as a cognitive, primarily social coping strategy. Our framework comprises a range of testable predictions, provides a new theoretical basis for future empirical investigations into photography, and has practical implications. We conclude that photography has become a human universal, which is based on context-sensitive mental predispositions and differentiates itself in the social and societal environment.

## Introduction

Photography is ubiquitous around the world, with the number of people taking and using personal photographs steadily increasing (Lee and Stewart, 2016; Canon, 2018). More than 90 percent of all photographs (henceforth *photos*) are taken with smartphones (Carrington, 2020), and more than half of the world's population uses smartphones or mobile phones to take, view, and share photos (Statista, 2019; Kemp, 2021). Smartphones integrate photography with many other functions, notably with access to the internet and social media (Smith, 2011; GSMA and NTT DOCOMO, 2014). This has rapidly shifted photography from an exclusive activity of socio-economically capable minorities toward engaging a majority of the world's 7.8 billion people.

We examined the question why people take, view, own, share, and use personal photos, and why photos are important to them. We consider the distribution of smartphone technology and social media a precondition for the sweeping success of photography, but insufficient to explain it, as not all smartphone features or technologies are widely used just because they are available. The technology to make audio-recordings, for example, has not been adopted by many people (Milgram, 1976). Although smartphones are capable of easily recording the voices of loved ones, conversations, the sounds of a birthday party, or of a strange city, people rarely use this function (GSMA and NTT DOCOMO, 2014; Lutter et al., 2017).

There is extensive research on the psychological bases of pictorial representation and art (e.g., Deacon, 2006; Donald, 2006; Dutton, 2009). No theory, however, has suggested an integrated psychological basis of the wide range of photography-related behaviors. Photography differs significantly from other visual representation techniques. The invention and further technical developments in photography have conveyed images with characteristics that drawings, paintings, maps, or plans do not have: (a) photos are realistic in a special way; (b) photos are produced by technical devices; (c) part of the information in photos is there by chance; (d) people tend to believe that what they see in photos really happened that way; and (e) photos can be created easily, quickly and effortlessly. We will describe these properties in more detail at the beginning of the following section.

Milgram (1976) assumed that taking and using photos conveys specific abilities, which can be best understood if cameras and photos are regarded as “evolutionary developments” (p. 7). We followed this approach and hypothesized that the urge to take, view, share, and use photos is based on *human nature* (Wilson, 2012; Kotrschal, 2019), i.e., on evolved context-sensitive predispositions and mechanisms, mainly rooted in the social domain. We examined this hypothesis on the basis of the four levels of Tinbergen (1963) to analyze and explain “natural” traits, i.e., those which evolved via the Darwinian processes. These levels relate to (1) the physiological mechanisms underlying a certain behavior, (2) it's ontogeny, (3) evolutionary history, and (4) adaptative value. This frame guided half a century of behavioral research and may be considered the research program of organismic biology in general (Bateson and Laland, 2013; Nesse, 2013).

We place photography in the context of the coherent theory of the evolution of life (Darwin, 1859, 1879/2004; Jablonka and Lamb, 2014) and human nature as an outcome of this evolution. The four levels proposed by Tinbergen are the theoretical and practical formulation of this context. Since there is only a single Darwinian theory of evolution, and culture is part of human nature (Jablonka and Lamb, 2014), the biological context should allow us to develop a unified explanation, coherent with contemporary knowledge particularly on the proximate mechanisms (i.e., current physiological mechanisms and their ontogeny). According to the four levels of Tinbergen, our central research questions are: What are the cognitive and physiological mechanisms underlying taking and using photos? How does taking and using photos develop ontogenetically?—which is important for understanding the development of inter-individual variation. What is the phylogenetic basis for photographic behavior? What may the functions and adaptative value of taking and using photos be? In this respect, a contribution of taking or using photos for survival and individual reproductive success may not be obvious in modern humans, but to qualify as an evolutionary function, the proof of a direct effect would not be needed. Rather, it would be sufficient to find a plausible positive effect on a person's social and mental well-being, which, in turn, on a population level, would entail a positive, supportive effect on societal and biological fitness.

Our aim was to create a theoretical framework, which describes why and in what way taking, viewing, sharing, and using personal photos are functional behaviors in terms of what is presently known about human nature. The development of this framework was based on the integration of available empirical findings on photography from multiple research areas with findings from biology, psychology, and neuroscience. We consider cultural and biological traits as closely interconnected and interacting in driving evolution and individual behavior (e.g., Jablonka and Lamb, 2014; Kotrschal, 2019). To the best of our knowledge, a similarly comprehensive integration of findings into a coherent theoretical framework has not been attempted before. Our framework generates a number of predictions about the specific characteristics of personal photos and photography-related behaviors, which can be tested through empirical investigations.

Based on our framework and data on the global availability of smartphones and social media, we intended to show that photography qualifies as a *human universal* (Murdock, 1945; Brown, 1991; Antweiler, 2016; Christakis, 2019; Kotrschal, 2019). The concept *human universal* is traditionally associated with traits, activities, characteristics, or institutions, which are observed in all cultures and societies worldwide, like social organization, cooking, language, music, or weapons (Brown, 1991). According to this view, photography would not be a human universal. Historically, photography is a new development and did not exist in the traditional societies described by ethnology. For traits or behaviors, which have only recently become universal, Brown (1991) introduced the term “‘new’ universals” (p. 50). He cited dogs, tobacco, metal tools, and plastic containers as likely examples. Hence, according to Brown's classification, photography is a “new universal.” We describe photography as a human universal, which is based on context-sensitive predispositions, which differentiates itself over ontogeny in the societal environment. Our evolutionary approach does not suggest categorizing photography as a stereotypic behavior based on “innate” dispositions. In line with the present concepts of human social behavior and human universals, we emphasize context-sensitivity, inter-individual variability and individual uniqueness of photography-related behaviors within the frame of the human *reaction norm* (Woltereck, 1909), as comparable, for example, with language or music.

## Materials and Methods

### Specific Characteristics of Photographs and Photography

Our focus is on *personal photography*, that is, on photography-related behaviors, including taking, viewing, sharing, and using photos, which are performed for personal reasons and without commercial intent (Chalfen, 1987; Kindberg et al., 2005). In particular, we refer to photography-related behaviors, which people perform immediately and voluntarily (spontaneously), without intentional preparation or planning beforehand. We specifically referred to characteristics of photos and behaviors related to photos, which other representational pictures and behaviors associated with them do not have:

#### Photos Are Realistic Images

An object depicted in a photo can share a large number of visual features with the object that was seen in the environment at a specific point in time from a specific location (Bradley and Lang, 2007). Because of this characteristic, photos are called *realistic* images (DeLoache et al., 1998). When individuals see a photo, a retinal image can be formed, which is similar to the image that would be formed if they saw the represented event or object in the environment in real life (Perrett et al., 1991). When investigating the neural bases of recognizing or categorizing objects (e.g., faces, bodies, sites, or objects), neuroscientists and cognition researchers often assumed that there is an equivalence between the photographic representation and the perceptible object in the environment and presented photos of objects as stimuli instead of the real objects in question. Important psychophysiological mechanisms underlying photography-related behaviors are related to the fact that photos of objects elicit reactions in certain areas of the brain similar to events, which are effectively seen in the environmeint.

#### Photos Are Produced by Technical Devices

Drawings and paintings can also be realistic images. In contrast to photos, the creation of drawings and paintings involves the hands of the artists who created them, and important visual characteristics resulted from the dispositions, ideas and decisions of these artists. Photos are created by technical devices, and viewers know this fact.

#### Part of the Information Came Into the Photo by Chance

The people who use cameras choose a certain perspective, a certain frame and a certain moment when they press the shutter button. Photographers use this selection to control the characteristics and meanings of photos. In complex natural scenes, photographers cannot control all of the information that gets into the photos. Some information comes into the pictures by chance (Talbot, 1844/2011). This is hardly the case with representative drawings or paintings.

#### People Assume They See Reality in Photos

People tend to believe that what they see in photos really happened that way—even if photos are posed, manipulated or forged (Wade et al., 2002; Nightingale et al., 2017). This phenomenon is still poorly understood. It is possibly related to the knowledge of the viewers that they see a picture that was produced by a technical apparatus. This knowledge could be linked to the assumption that the picture is little affected by the personal attitudes and intentions of the person who made it (Miller, 1973; Gu and Han, 2007).

#### Photos Can Be Created and Understood Easily, Quickly, and Effortlessly

Unlike drawings, paintings, maps, or plans, photos can be created easily, quickly and effortlessly. Three-year-old children can take informative and expressive photos (Magnusson, 2018). Without complex knowledge or skills, people can take photos that they and other people find excellent (De Looper, 2016). Complex events represented by photos are quickly and easily understood. A single quick glance is enough for viewers to understand, for example, an interaction between two individuals (Hafri et al., 2013).

Taking, sharing, and using photos are not behaviors, which have all of a sudden appeared as something completely new and an emergent property of culture. We hypothesized that they are deeply rooted in organismic and cultural evolution. The basic cognitive and physiological factors underlying photography-related behaviors are common to all people. Some of these factors may vary relatively little between individuals, but others, for example, related to individual personality structure may show great inter-individual variability. But even such a pronounced inter-individual variability is far from random, as much of ontogeny seems to depend on context-sensitive human dispositions (e.g., Jablonka and Lamb, 2014; Kotrschal, 2019). Such dispositions are the result of non-random interactions between genes, epigenetics, and the social and societal environments during ontogeny. They frame the way people tend to take, view, share, and use photos.

### Empirical Data and Findings on Photography-Related Behaviors

Empirical data and findings on taking, viewing, recognizing, sharing, and using photos come from a variety of disciplines, such as psychology, neuroscience, human-computer interaction, and anthropology. In analyzing the questions on the level of the cognitive and physiological mechanisms underlying photography-related behaviors, we referred to studies that examined the following questions: Which cognitive processes in the brain play a special role in photographing (Barasch et al., 2017; Blitch, 2017)? How do people's brain responses to photos they have taken themselves differ from their responses to photos taken by others (Sellen et al., 2007; St. Jacques et al., 2011; Diefenbach and Christoforakos, 2017)? Which brain responses do photos elicit in which viewers see a person with whom they are connected through a close emotional relationship (Bartels and Zeki, 2004; Gobbini et al., 2004; Leibenluft et al., 2004; Master et al., 2009; Eisenberger et al., 2011)? Which brain responses do photos evoke in which viewers see themselves (Devue et al., 2007; Butler et al., 2012)? Which neural processes form the basis for viewers to find a picture beautiful or ugly (Kawabata and Zeki, 2004; Jacobs et al., 2012)?

To describe the ontogenesis of photography-related behaviors, we refer to studies that examined the development of the ability to recognize the representational properties of photos (DeLoache et al., 1998; for review, see Bovet and Vauclair, 2000), as well as to studies, which examined the age at which children start taking photos and for what purposes they use cameras (Mäkelä et al., 2000; Sharples et al., 2003; GSMA and NTT DOCOMO, 2014).

In analyzing the evolutionary roots of photography-related behaviors, we refer to studies of the ability of non-human primates and other animals to recognize objects depicted in photos (Bovet and Vauclair, 2000; Kano and Tomonaga, 2009; Aust and Huber, 2010). Information was also provided by investigations into the question how people develop the ability to recognize objects pictured in photographs (Deregowski et al., 1972; Miller, 1973; Bovet and Vauclair, 2000).

Table 1 briefly summarizes some of the research that will be used to analyze the level related to the adaptative value of photography-related behaviors. Every single referenced study provides a number of answers that are not always consistent with the answers from the other studies. The answers given are therefore rather examples of content to which we refer in the article than representative information.

**Table 1:** Questions and studies used to analyze the adaptative value of photography-related behaviors.

**Questions**	**Studies**	**Answers**
What do people photograph?	Crandall et al., 2009; Hu et al., 2014; De Looper, 2016	Family, friends, themselves, pets, activities, celebrations, food, fashion, nature, famous places, and landmarks
In which situations and for what purposes do people take photos?	Bourdieu, 1965; Chalfen, 1987; Kindberg et al., 2005; Barasch et al., 2014	People take photos of significant events for personal and/or social purposes
Does the emotional state of people influence whether and what they photograph?	Chalfen, 1987; Gillet et al., 2016; Diefenbach and Christoforakos, 2017	Photographing is influenced by states in which people experience a pleasant event, which is also related to the processing of uncertainty
How does taking photos influence how the photographers and the photographed individuals experience a situation?	Burgess et al., 2000; Mols et al., 2015; Diehl et al., 2016	Photographing increases the pleasant experience of a situation and the feeling of social connectedness
How does photographing an event affect how well photographers later remember this event?	Henkel, 2014; Barasch et al., 2017; Blitch, 2017; Jain and Mavani, 2017	Photographers tend to remember better visual features of the photographed event later, but less non-visual features
What are the characteristics of photos that people consider successful or “good”?	Kirk et al., 2006; Bakhshi et al., 2015	The photos have enough desirable visual and/or representational characteristics
What do people do with the photos they have taken?	Schiano et al., 2002; Kindberg et al., 2005; Kirk et al., 2006; Broekhuijsen et al., 2017	People keep photos, edit some, share them, use them for social purposes and/or autobiographical remembering
How do people use the photos they have taken on online social networking services, and does seeing photos in social media affect the emotional state of the viewers?	Krämer and Winter, 2008; Hu et al., 2014; Lee et al., 2015; Malik et al., 2016; Pittman and Reich, 2016; RSPH and YHM, 2017	People use photos to evoke attention and engagement, and to share important information. Seeing the photos affects emotional arousal and evaluations
How do people use photos in connection with courtship and mating behaviors?	Piazza and Bering, 2009; Sedgewick et al., 2017; Gale and Lewis, 2020	People create and show photos of themselves in which they are represented in the way they want to be seen by potential sexual or romantic partners
What importance do personal photos have for families?	Csikszentmihalyi and Rochberg-Halton, 1981; Petrelli and Whittaker, 2010; Whittaker et al., 2010; Frohlich et al., 2013	Photos are one of the most precious possessions of families. Photos symbolize the roots, importance, or meaning of a family

## Results: the Four Levels of Tinbergen (1963) As a Theory Frame

### Psychophysiological Mechanisms Underlying Photography-Related Behaviors

**Figure 1:**
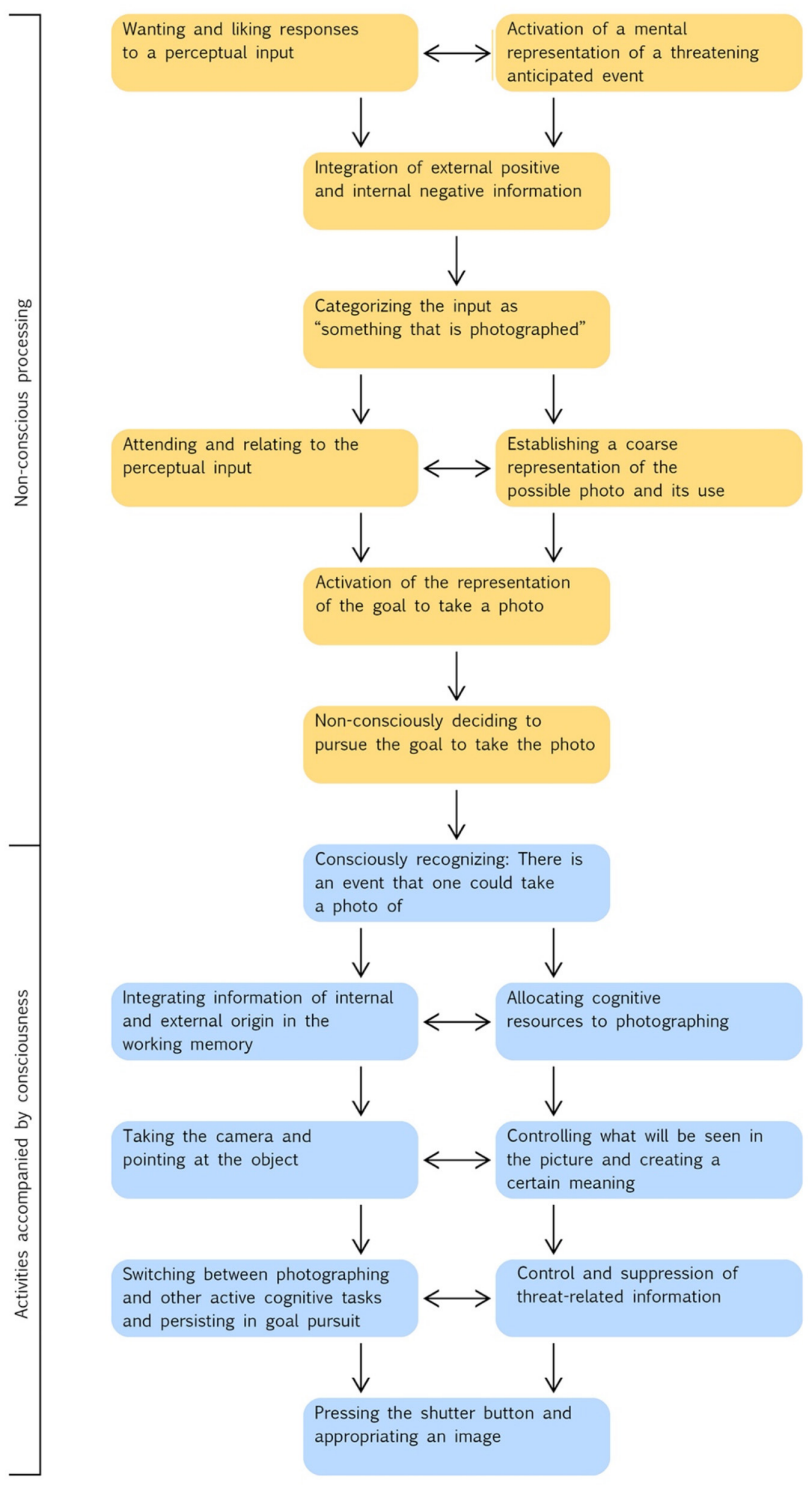
Hypothetical process model including the major processes and activities that occur in an individual who engages in taking a personal photo. The place within the sequence where the processes and activities are located indicates either when they occur or when they first occur. In order to keep the presentation clear, possible feedback effects of activities on the antecedent steps are not shown. Downward arrows mean “then occurs”; a horizontal arrow means “interacts with”.

Researchers have used photos as stimuli. Thus, quite some knowledge on the psychophysiological mechanisms involved in recognizing and viewing photos has accumulated, but experimental research on the mechanisms involved in taking photos is essentially lacking (except for Blitch, 2017). The success of photography, however, is primarily related to features of taking photos (Krämer and Winter, 2008; Hu et al., 2014; Lee et al., 2015; De Looper, 2016; Malik et al., 2016; Carrington, 2020). These include various activities and outcomes. These activities are, for example, associated with relating to individuals or objects as well as creating and appropriating images of them and their desirable properties. Outcomes may be associated with a sense of control and efficacy. The rapid global spread of photography was not driven by new opportunities to get, acquire, or exchange photos taken by other people, but mainly by the increased availability of inexpensive cameras, particularly smartphones, and opportunities to share one's own photos electronically. For this reason, we address in this section the specific mechanisms that form the neural basis of taking personal photos. The following description of the processing steps in taking pictures corresponds to hypothetical predictions. We mainly employ findings on processes in primates including humans from various contexts, which can be related to the psychophysiological mechanisms involved in taking photos. Figure 1 shows a hypothetical model including the major steps of taking a photo.

#### Initial Steps in Taking Photos

The first steps in taking a photo do not involve conscious awareness (Custers and Aarts, 2010). A mother, for example, responds spontaneously to the happy expression on the face of her 6-year-old son at his birthday party, or a hiker responds to the overwhelming panorama at a mountain top. In these examples, the perceptual input activates a fast, low-level system of subcortical structures related to affective processing (Baxter and Murray, 2002; Pourtois et al., 2013), including neurons responding to the visual information and others responding to relevance and information related to primary (evolutionarily developed) or individually acquired reward value. Some of these structures project to the midbrain dopaminergic system (Dommett et al., 2005; Schultz, 2006). In turn, dopaminergic projections from the ventral tegmental area (VTA) in the midbrain reach the ventral striatum, including the nucleus accumbens (NAcc), amygdala, hippocampus and other areas of the mesolimbic system (Berridge and Robinson, 1998; Alcaro et al., 2007), which functions as the central neural basis for approach and motivation. This mesolimbic system overlaps with the social behavior network in the brain, responsible for the control of social behavior (Goodson, 2005; O'Connell and Hofmann, 2011).

The activity of the dopaminergic neurons in the brain of the mother who sees her happy son corresponds to a “wanting” reaction (Berridge and Robinson, 1998). It makes her son's excited face salient and attractive. The fact that the mother *likes* what she perceives may be related to the release and processing of endogenous opioids (Panksepp, 1998; Kringelbach and Berridge, 2009; Hsu et al., 2013). Whereas dopamine conveys motivational incentives, endogenous opioids convey “liking,” but also have a calming effect and reduce neural responses to pain, stress and anxiety (Carter, 1998; De Kloet et al., 2005).

In everyday life, people usually take photos of pleasant events (Chalfen, 1987; Sharples et al., 2003; Hu et al., 2014). We assume, however, that the motivation to take a picture is often also related to the activation of a mental representation of a negative context, which is processed non-consciously. In our example, this negative context would be that the mother knows that her son celebrates his last birthday party before entering school. As her own mental representations of school are ambivalent, she develops an anticipatory concern regarding the situation of her son, which is threatening and creates mental stress (Ulrich-Lai and Herman, 2009). Representations of such threats correlate particularly with activities in the amygdala (Baxter and Murray, 2002; Pourtois et al., 2013), triggering a cascade of adaptive neural and neuroendocrine reactions (De Kloet et al., 2005; Schiller et al., 2008; Ulrich-Lai and Herman, 2009; Hostinar et al., 2014). They include the activation of the stress systems leading to an increase in excitement and alertness.

Hence, we suggest that two conflicting representations are activated in the mother's brain, each associated with a different behavioral response than the other. The mother needs to mobilize cognitive and behavioral resources to be able to balance the two possible meanings and reactions, which in essence employ different parts of her brain. The anterior cingulate cortex (ACC) plays an important role in this. The ACC lies inside the frontal cortex, where it extends around the dorsal side of the corpus callosum, the nerve tract that connects the two cerebral hemispheres. It integrates and organizes emotional and cognitive information related to coping with pain, fear, anxiety, and stress, and potential motor responses, and is a major neural basis of cognitive control (Bush et al., 2000; Shenhav et al., 2013). Cognitive control is defined as regulating reactions to pieces of information that are in conflict with one another and in which automated processing may lead to errors (Miller and Cohen, 2001). The goal of cognitive control is to integrate conflicting information into representations that support appropriate behavioral decisions.

The mother's medial prefrontal cortex (mPFC) signals that there is something out there that offers the opportunity to collect or appropriate something valuable—mPFC is a central part of the neural basis of appropriating or collecting something (Anderson et al., 2005; Turk et al., 2011). Based on the dopaminergic processes involved, the motivation for appropriating something can be very strong: mPF and ACC have the greatest densities of dopaminergic projections from the midbrain of all areas in the cortex (Williams and Goldman-Rakic, 1993; Cohen et al., 2002).

Based on her photography-related knowledge, the mother categorizes what she perceives as “something that is photographed.” What is going on out there, could enable her to create a valuable picture. According to the assumptions of *Event Cognition* (Newtson, 1973; Zacks et al., 2001), “a children's birthday party” is not represented in the mother as a continuous, uniform event, but in the form of a few interconnected discrete units or steps, such as welcoming the guests, eating the birthday cake, blowing out the candles, and so on. The mother has detected that such a discrete step of the party has occurred. A photo of it could represent much of her son's birthday party. Activation patterns in prefrontal and hippocampal areas switch on photography-related memory contents that are connected to one another and retained in various locations widely spread over the cerebral cortex (Tonegawa et al., 2015). Context and scene are associated with possible outcomes of taking a photo with a smartphone camera, including a coarse anticipatory representation of the possible photo and its use.

Still without the involvement of conscious processes, the representation of the goal to take a photo is activated in structures of the mother's anterior prefrontal Cortex (PFC) (Soon et al., 2008; Custers and Aarts, 2010). Processes in OFC, mPFC, ACC, and ventral striatum analyze whether the goal to take a photo can be achieved in the given situation, and whether it is worth the effort. The result is the decision that the photo is worth the effort.

#### Steps Accompanied by Conscious Awareness

For taking the photo, representations from different explicit and implicit memory and processing systems must be integrated. Our mother is now consciously recognizing (Dehaene and Naccache, 2001; Damasio, 2010) that she is perceiving something that might be worth photographing. She takes her smartphone and points the camera at her son, who is surrounded by friends. She controls what will be seen in the picture. OFC, ACC, amygdala, and the anterior insula build the neural bases of various valuation, filtering, ordering and decision processes (Hsu et al., 2005). The mother's working memory (Baddeley and Hitch, 1974; Miller and Cohen, 2001) processes, maintains and integrates different pieces of information of internal and external origin.

The mother takes a photo of her son, a person with whom she is connected through a close positive emotional relationship. Seeing him activates areas in the mother's brain that have a high density of the peptide hormones oxytocin and vasopressin (Bartels and Zeki, 2004). Oxytocin and vasopressin are produced in the hypothalamic Nucleus preopticus (NPO), stored in pituitary, and are involved in the development and maintenance of close selective social relationships (Carter, 1998; Panksepp, 1998; Scheele et al., 2013; Hostinar et al., 2014). They also support the control and suppression of threat-related information (Nelson and Panksepp, 1998; Donaldson and Young, 2008; Scheele et al., 2013). Particularly oxytocin is involved in the development and maintenance of close selective social relationships or attachment and conveys the feeling of social connectedness (Carter, 1998; Panksepp, 1998; Scheele et al., 2013; Hostinar et al., 2014). Both hormones are associated with activating the mesolimbic reward system (Donaldson and Young, 2008). Oxytocin release correlates with opioid activities, reduces stress and thereby causes a calming effect (Nelson and Panksepp, 1998). In fact, there is a strong antagonism between oxytocin release and glucocorticoids synthesis, i.e., metabolic hormones that are produced and released in response to stressors (Carter, 1998; Hostinar et al., 2014; Preckel et al., 2015).

The mother's vmPFC assigns a positive value to the neural representations of the situation, photographing in general, and the intended photo in particular. On a non-conscious processing level, however, the anticipatory representation of the threat of her son's potentially negative experiences at school is still effective. This threat is primarily processed in the amygdala, but the mother's vmPFC projects into the amygdala and, thereby, inhibits its activity, which reduces fear and anxiety (Andolina et al., 2013; Hostinar et al., 2014). In addition, vmPFC, OFC and ACC project to the hypothalamus and reduce the activity of the mother's stress systems (Ulrich-Lai and Herman, 2009; Hostinar et al., 2014). Her implicit processing mechanisms suggest that she can now safely ignore the threat (Schiller et al., 2008).

When she recognizes a sufficient correspondence between the characteristics of the picture on the smartphone display and the mental representation of the desired photo, she presses the shutter button. She creates a permanent external picture of her son in a particular context, a representational digital object, which she possesses and can share with others. An important part of the value the picture has for her is related to the fact that she has created it herself. Actually, people can reliably distinguish between photos that they have taken themselves and photos taken by others (Sellen et al., 2007; St. Jacques et al., 2011).

A mountain hiker who discovers something she wants to photograph may have a different experience than a mother at her son's birthday party. She likes to hike alone and enjoys nature and silence. When looking at the mountain landscape, the anticipation of a longer period of non-self-chosen solitude has been activated. The hiker can take a picture, which will allow her to share her experience with her friends. Unlike our example mother, the hiker has more time for taking the picture, because the landscape does not change as quickly as social situations at a party. The hiker can use this time for creatively composing a photo, which will be different from ordinary photos depicting similar landscapes and which the viewers will find beautiful, useful, or important (Thagard and Stewart, 2011; Ellamil et al., 2012). She associates and integrates the incoming visual information with certain conceptual and emotional categories as well as with internal representations of existing extraordinary landscape pictures. The neural bases of these operations include structures of two cortical networks that are usually not active at the same time. One of these networks is activated when people focus their attention on external stimuli, the other network when attention is focused on thoughts, memories or imagery (De Pisapia et al., 2016).

### Ontogeny of Recognizing, Taking, and Using Photos

Human babies recognize certain photos at an age of 3 months or even earlier (for review, see Bovet and Vauclair, 2000). In a cross-cultural study, DeLoache et al. (1998) showed that 9 months old babies treated pictured objects as if they were real objects, explored them with their hands, tried to touch them, or to take them out of the picture. At the age of 19 months, human children understood that pictures are both concrete real objects, but also representations of other objects. From about 1-year of age, children begin to create traces on two-dimensional surfaces with suitable materials (Thomas and Silk, 1990; Wright, 2010). At the age of two, children begin to name the meanings of their drawings or paintings. They also know that pictures are made with specific intentions to represent objects or events (Preissler and Bloom, 2008). Children aged 3- to 4-years know what properties of pictures are helpful if they are used to convey ideas of objects to other people, and that there are better and worse pictures for this purpose (Allen et al., 2010). They know that pictures containing a lot of visual details are best used to tell others what objects look like.

Many children like to draw. As much as they develop joy and zeal in drawing, they usually have little interest in owning the pictures as soon as they are done (Thomas and Silk, 1990; Cox, 2005; Cherney et al., 2006; Wright, 2010). If they have mastered a special pictorial challenge, they proudly show their picture and look at it together with others, but they do not go for drawings they made the week or the month before to look at them again. The fascination lies in the activity of drawing itself, in experiencing the ability to create a picture with a certain meaning—and to use this to relate to others (Cox, 2005; Wright, 2010). The early ontogenetic development of competences related to producing and using representational pictures happens in the social environment, usually the family. The family is also one of the most productive places of personal photography (Chalfen, 1987; Petrelli and Whittaker, 2010). The most successful photography exhibition of all time even had “family” in its title: The Family of Man (1955).

Children see the photos their parents keep in photo albums, photo books, boxes or computer folders. The photos of the ancestors—and their actions, experiences, relationships, occupations, and possessions—that a family owns can give children a sense of social belonging, societal significance, and security (Csikszentmihalyi and Rochberg-Halton, 1981; Chalfen, 1987; Petrelli and Whittaker, 2010). These photos are heritable assets of knowledge. They are usually linked to oral or written information, which shows and tells to whom the children belong and whom they can trust. Through mechanisms of social learning, family traditions of photography emerge (Mäkelä et al., 2000; Sharples et al., 2003; Petrelli and Whittaker, 2010). Children get to know certain ways of using cameras and photos early on (Mäkelä et al., 2000; Sharples et al., 2003). They experience how their mother or father reacts to certain events by taking photos—usually positive, which supports this behavior via positive reinforcement learning. Children also realize that taking, viewing, and sharing photos is repeatedly done in certain social contexts, for example at a birthday party, graduation, or wedding. They learn that photographers keep some pictures and discard others and may shape their own taste along this.

Many children start taking photos themselves at preschool age (Sharples et al., 2003; Magnusson, 2018). Seven- to 15-year-olds take and use photos in connection with the playful and explorative use of electronic devices (Mäkelä et al., 2000; Sharples et al., 2003). They use cameras and photos for joking, like making faces or adopting funny poses, for expressing feelings, or telling stories. According to an international survey, 81% of the 8- to 18-year-olds in Algeria, Egypt, Iraq, and Saudi Arabia used a mobile phone in 2013 (GSMA and NTT DOCOMO, 2014). Most of the children got their own mobile phone between 10- and 12-years of age and 55% had access to the internet. The features most used by children and adolescents were cameras (91%), followed by music players and video players. Many young people in their teens and early twenties take and use photos to create a sense of self and an identity (Schiano et al., 2002; RSPH and YHM, 2017). Social media provides them with a platform where they can use photos to express different characteristics of themselves and to experience other people's reactions (Krämer and Winter, 2008; Hu et al., 2014; Lee et al., 2015). Photos of family members and pals are especially important for people (Mäkelä et al., 2000; Sharples et al., 2003), and the value of these photos increases with the age of their owners (Csikszentmihalyi and Rochberg-Halton, 1981).

### Evolutionary Roots of Taking and Using Photos

**Figure 2:**
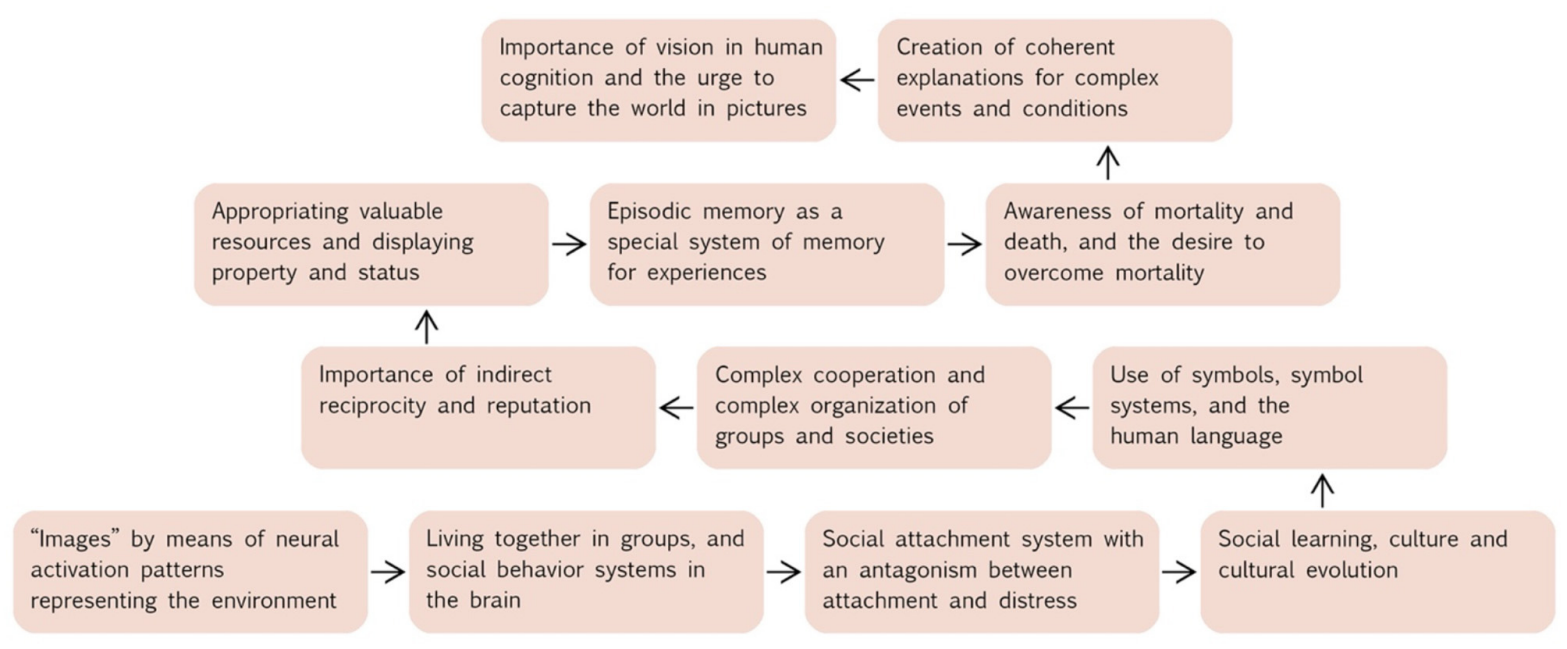
Schematic illustration of the building blocks that are part of the evolved nature of modern humans and form the basis of the importance of photos for people. The arrows show the order in which we describe the building blocks in our model.

Why do photos have the characteristics they have? Why are they important for people all over the world? Which meanings can almost only be represented and communicated through photos Kislinger, 2021), and which cannot? In this section, we will refer to cognitive and social building blocks, which are part of the evolved nature of modern humans and suggest answers to these questions. Figure 2 presents an overview of the evolutionary building blocks that underlie the success of photography to which we refer.

#### Vision as a Central Element of Human Cognition

People take and use photos to represent important events in the environment. Representing features of the environment with survival value is one of the core functions of central nervous systems (CNSs) since they exist. Organisms developed sensory organs, which react to relevant physical and chemical events in the environment, as well as neurons, that is, cells capable of receiving, generating, and transducing signals for internal communication and for relating to the environment (Butler and Hodos, 2005; Gregory, 2008). By means of neural activation patterns, organisms have used “images” for hundreds of millions of years (Damasio, 2010)—as representations of the environment enhancing predictability in interaction with this environment and, thus, survival. Although it is hard to imagine how a jellyfish with its dispersed nervous system should be able to form an image-like mental representation, the fact that its body responds to stimuli in a coordinated and adaptive way at least hints at such a possibility.

Mammals evolved out of mainly visually oriented reptiles (Northcutt, 2011; Striedter, 2020). During their first 100 million years of evolution, however, the reign of dinosaurs forced them underground or into a nocturnal lifestyle. This led to a reduction in visual orientation, while olfaction and hearing were optimized. Within the modern mammals we see a full reinstatement of trichromatic vision only in the primates, while most other mammals remain bi-chromatic as an adaptation to being active at dusk and dawn and at night. Due to specific properties of their central nervous systems, including retinae, primates can extract a broad range of information from the properties of light and its reflections in the physical world (Felleman and Van Essen, 1991; Gollisch and Meister, 2010). Vision is a central component of human cognition. Visual content dominates, for example, perception, memory, imagining, and dreaming (Posner et al., 1976; Zimmermann, 1989).

#### Living Together in Groups and Social Attachment

People primarily take photos of other people, especially people to whom they are emotionally connected (Chalfen, 1987; Hu et al., 2014; Lee et al., 2015). Processing stimuli with social significance has a long evolutionary history (Wilson, 1984, 2012). The tegmental and diencephalic parts of the brains of birds, bony fish, and mammals feature an evolutionary extremely conservative—hence homologous—social behavior network (Goodson, 2005). This regulates social recognizing, mating, parental behavior, persistent bonding, expressive behavior, aggression, and responses to social stressors. Primates inherited this network virtually unchanged in structure and function from their ancestors. The primate ancestors of humans established close relationships with other individuals in their groups who were not reproductive partners or relatives (De Waal and Brosnan, 2006; Wilson, 2012). Social cohesion improved the ability of individuals and groups to adapt to variable environments, to survive and to reproduce. Living together in groups affected both behavior and cognition. In primates, the social domain hosts a substantial part of the motivation to orient to and perceive stimuli, and to carry out certain behaviors and actions. Important and mutually linked social behavior systems are *attachment* and *care*, that is, a close selective emotional connection with another individual—the caregiving attachment figure, or the other way round, the attached dependent (Bowlby, 1974; Carter, 1998; Panksepp, 1998). There is a strong antagonistic interaction between the feelings of safe attachment and distress (Panksepp, 1998). Threatening or stressful situations elicit the desire for social closeness, and societal cohesion increases in times of crisis. Support by an attachment figure provides a sense of security and calmness. Conversely, being isolated from attachment figures or other socially supportive individuals is perceived as a potential threat. This antagonistic interaction is relevant in terms of photography-related behaviors. Photos of attachment figures or of the attached dependents convey important potentials. In experiments, for example, merely seeing the photo of an attachment figure reduced physical pain as effectively as the actual closeness to that person (Master et al., 2009; Eisenberger et al., 2011).

#### Social Learning, Cultural Evolution, and Symbol Systems

An important ability of animals living in groups is profiting from experiences or interactions with other individuals, called *social learning* (Richerson et al., 2010; Jablonka and Lamb, 2014). A system of characteristic behavior patterns and preferences, which are socially passed on through generations, is referred to as culture and its gradual change as *cultural evolution* (Jablonka and Lamb, 2014). Cultural phenomena have probably played a greater role in human evolution than in any of the other animals showing cultural diversification (for example wolves or orcas) ever since the common ancestors of humans and chimpanzees (Richerson et al., 2010; Whiten, 2011).

Social learning is the base for tradition forming and transferring information via culture. The ancestors of humans used gestures, vocalizations, and found objects as signs for something that was not currently present in the environment to communicate with others (Seyfarth et al., 2005; Deacon, 2006; Arbib et al., 2008). Over many generations, groups gradually developed a complex system of gestural and vocal signs, as well as rules specifying how these signs were to be combined into larger units of meaning (Seyfarth et al., 2005; Arbib et al., 2008). As a crucial step in human evolution, humans began to use *symbols*, this is, signs that represent meanings based on rules and conventions. Symbols are part of an evolved cultural system, which regulates the relationships between individual signs and indicates how they are combined to represent units of meaning (Jablonka and Lamb, 2014). The use of symbols for organizing and conveying information was a crucial step in human evolution. Human symbols are considered as discrete dimensions of inheritance and evolution which interact with genetic evolution. People developed *systems of symbols* to represent and communicate knowledge, rules and ideas (Jablonka and Lamb, 2014; Tomasello, 2014). Language became the most important symbol system, likely also pushing brain development. Cultural evolution and genetic evolution interacted and led to a positive feedback selection between cognitive mechanisms, language, and social skills (Deacon, 1997; Jablonka and Lamb, 2014).

#### Cooperation, Property, Status, Reputation, Courtship, and Mating

Among the evolutionary mechanisms, which favored cooperation in groups, direct and indirect reciprocity appear to be particularly relevant (Nowak, 2006). These mechanisms are also relevant in terms of taking and using photos. Direct reciprocity is effective when two individuals encounter each other repeatedly: one cooperates assuming that the other one will reciprocate later. Cooperation, hence, benefits both. The mechanism of indirect reciprocity explains cooperation in situations where one individual helps another individual whom the individual may not meet again or from whom no help is expected. This can still pay off, if the helpful behavior is observed by other group members. Indirect reciprocity describes the benefit of an altruistic act for the helping individual, which spreads via gossip or other information. In this detour, the helping individual acquires the reputation of being “generous,” i.e., able and willing to cooperate. This reputation supports access to resources and reproductive success (Nowak and Sigmund, 2005; Nowak, 2006). With the evolution of complex language—and later with the distribution of photos—the subset of a population that could receive information about the cooperative potential of an individual tremendously increases as compared to the number of people able to directly observe an individual's behavior. Photography and social networking services on the internet have increased the potential audience enormously.

In human societies, it is generally advantageous to regulate resource use and ownership through rules or conventions in order to avoid costly redundant conflicts (Stake, 2004). Depending on socio-economic background, people have developed specific rules about the appropriation of things as well as about the retention and distribution of property (Stake, 2004). Many animals appropriate things and retain them (Stake, 2004). Property-related experiences and behaviors are based on specific neural substrates, especially in the frontal cortex (Anderson et al., 2005; Turk et al., 2011). The brain structures involved are particularly rich in dopamine receptors. The acquisition of property is accordingly associated with strong motivation. When individuals acquire and possess valuable resources, it may also be beneficial to their status within their groups, or may even be to the benefit and status of these groups (Brown, 1991; Van Vugt and Tybur, 2016). Much of human social complexity is about status and prestige. This modulates, in turn, individual access to resources in a social dynamic between cooperation and competition (Nowak and Sigmund, 2005; Van Vugt and Tybur, 2016). Individuals can display their property and signal that they have a certain status within the social and cultural hierarchies of their group or society. To communicate this status, individuals may use symbols of their possessions. Individuals can also share their resources with others, be generous or even wasteful with their possessions to increase their prestige and, ultimately, their reproductive success (Buss and Schmitt, 1993). In women and men, the acquisition, retention, and use of resources or possession have specific characteristics (Brown, 1991; Buss and Schmitt, 1993).

Photography has provided people with effective means to signal their social status to a large audience (Krämer and Winter, 2008; Piazza and Bering, 2009). Distributing selfies with famous people or in front of famous sights, for example, is motivated by telling others about one's own potential to meet these famous people or to travel, and to communicate one's own interests and attitudes (Krämer and Winter, 2008; Diefenbach and Christoforakos, 2017). Many people also take and share status-relevant photos of themselves with their “belongings,” such as house, car, boat, their beautiful partner, or children (De Looper, 2016; Jain and Mavani, 2017). Empirical data suggest that people also use photos for enhancing their mate value in the minds of potential romantic or sexual partners (Piazza and Bering, 2009; Smith, 2016; Hobbs et al., 2017; Sedgewick et al., 2017; Gale and Lewis, 2020; Kemp, 2021; Morris, 2021). We will discuss this in more detail in the section on the functions of photography.

#### Memory and the Urge to Create Coherent Explanations for Events and Conditions

Humans improved their ability to use language to categorize behaviors, events, objects and states. They developed a special system of comprehensive memory for experiences, including social, called episodic memory (Tulving, 2005). Thereby, experiences of “what,” “with whom,” “when,” and “how it felt” are integrated in a way that individuals can consciously access their stored representations and have a comprehensive awareness of their own life as related to others. With the ability to represent, process, and communicate past and future, as well as possible or imagined events through language, came the urge to explain what happens in the world, to interpret the past and to predict the future (Pettitt, 2011). Humans developed an awareness of mortality, thinking about death, and the desire to overcome mortality. The earliest burial sites found with material traces of ritual practices are around 100,000-years old (Pettitt, 2011; Wilson, 2012). The desire for extending one's effectiveness beyond lifespan could also play a role in taking pictures (Csikszentmihalyi and Rochberg-Halton, 1981; Chalfen, 1987). Many people retain photos of ancestors in a respectful way, in the implicit understanding that their descendants will do the same. This is reminiscent of animistic cultures, where identity and existence of people are deeply rooted in cults around ancestors (Frazer, 1911; Bird-David, 1999).

When taking a photo of another person, the photographer not just appropriates a picture of the light reflections from this person, but also of the visual, behaviorally relevant signals that this person emits at that particular moment. This may be part of the reason why many people consider appropriating a picture of a person to have “magical” (Frazer, 1911; Kittredge, 1929) or “animistic” (Bird-David, 1999; Harvey, 2005) properties. The term “animistic” refers to the belief that not only humans, but also animals, plants, lakes, mountains, etc. have souls and are animated (Harvey, 2005). With taking a picture of a certain person her or his personality and even “soul” may be captured, and the owner of this picture can change the condition of the pictured person—with potentially negative consequences (Hetherwick, 1902; Frazer, 1911; Hocart, 1922). Image magic has a long tradition going back far into human prehistory (Kittredge, 1929). Today, there is an ongoing struggle for legal regulation of the protection of one's image as part of personal rights and property rights, indicating that personal images still retain their special private status. Even on a rational base, the power that is conveyed by taking, owning, and using photos [e.g., Regulation (EU), 2016], is still a delicate topic in modern Western societies.

Language enables humans to integrate a huge amount of information into meaningful contexts and to create explanations of events in which these events appear ordered and understandable toward a goal, rather than meaningless, accidental and pointless (Kahneman, 2011). In addition to language, an evolutionarily older cognitive system remained (Evans, 2008; Kahneman, 2011), providing quick reactions to relevant events in the environment on the basis of minimal sensory information, for example, via faces with emotional expressions or expressive body poses (Kislinger, 2021). Certain events depicted in photos cause activations of evolutionarily old brain structures, like superior colliculus, pulvinar, and amygdala (Morris et al., 1999; Van Le et al., 2013; Almeida et al., 2015). Objects and events pictured in photos are not only recognized by humans, but by many other species (Bovet and Vauclair, 2000; Kano and Tomonaga, 2009). In some cases, the last common ancestor of humans and a species in question lived long ago, e.g., 220 million years in the case of pigeons (Aust and Huber, 2010). This either hints at an ancient ability shared via phylogenetic inheritance (homology) or at parallel evolution (analogy).

### Functions and Adaptative Value of Photography

A “function” of a behavior describes a specific contribution of the individual expression of this behavior to survival and reproductive success (Jablonka and Lamb, 2014). Photography-related behaviors touch the evolutionary functional domains of well-being and social connectedness, which are at the core of human nature. These behaviors will therefore, directly or indirectly, relate to potential individual societal and—ultimately—reproductive success. We suggest that taking, owning, viewing, sharing, and using photos provide a specific and effective strategy for coping with complex environments fraught with uncertainty. Photography as a coping-strategy comprises four core domains: (1) making sense, (2) appropriating an image, (3) establishing and supporting social connectedness, and (4) courtship and mating. These four domains can be involved in different photography-related behaviors to different degrees.

#### Making Sense

“Making sense” plays a role in many photography-related behaviors (Harrison, 2002; Frohlich et al., 2013); it is particularly evident in the taking of photos (Chalfen, 1987; Gillet et al., 2016). Thereby, people assign a certain cause to an event—that is, they create an explanation for why this event occurs—or a certain meaningful order, which is consistent with a goal. The 6-year-old birthday boy from our example above laughs because he gets along well with other children, and other people want him to be happy. The photo of the hiker shows that being alone on a mountain top is great, because it gives one a deep personal feeling for nature, which still can be shared with friends via a picture. According to our framework, people build mental representations, which make an event understandable. As a consequence, the future course of the event appears predictable and controllable. Taking photos allows making sense immediately and intuitively, without the involvement of complex reasoning.

Sharing and viewing photos can also be used for making sense of events. The photo of a family reunion can show a group of laughing people who relate to each other in a friendly and nice way, even if a heated argument broke out at this meeting, which may have led to long-term insults and resentments. Particularly, people who were at this meeting can look at this photo to reassure themselves that, despite certain controversy, things are fine and people like each other. This is supported by the propensity of viewers to assume that what they see in photos reflects reality. Understanding photos does not require the mastery of a particular language, complex cultural knowledge, or elaborate thinking. A single photo can give a fairly comprehensive idea of an event—possibly better than any verbal description: “a picture is worth a thousand words” (The Post-Standard, 1911).

#### Appropriating an Image

The domain “appropriating an image” is related to the fact that people gain permanent access to valuable information by taking, sharing, or getting a photo—most frequently related to social relationships. Photographers relate to an event or object through the camera, select certain properties of this object and the scene in which it is contained, and create a focus. From the flow of the object's appearances, which they perceive over a certain period of time, they extract a single picture and fix it. It represents only a small fraction of the sensory information that is available in that situation. By selecting and organizing the information, which they include in the picture, they interpret the object or event. If they have managed to create the picture with the intended meaning, this success conveys the experience of effectiveness and competence (Krämer and Winter, 2008). This experience reduces emotional arousal and physiological stress responses to potential threats and supports coping with them (Bandura, 1997). A man looking out the window of the plane that is taking off, for example, may take a number of photos, thereby potentially also coping with his fear of flying. Taking photos may help to maintain control in a potentially stressful situation.

#### Establishing and Supporting Social Connectedness

Seeing an important person in a photo allows the viewer to relate emotionally to this person, although she or he is absent or may have passed away. Photos of their own children, parents, or romantic partners are particularly important to people (Bartels and Zeki, 2004; Gobbini et al., 2004; Leibenluft et al., 2004; Petrelli and Whittaker, 2010; Hu et al., 2014). Photos of loved ones enable people to feel close to them, provide a sense of security and calmness and reduce the sensation of pain (Master et al., 2009; Eisenberger et al., 2011). Photos, to some extent, can substitute for physical closeness. Viewing, owning, or sharing photos of family members or ancestors support developing cultural and genealogical roots (Csikszentmihalyi and Rochberg-Halton, 1981; Petrelli and Whittaker, 2010). Hence, taking and using photos relates to establishing, maintaining and strengthening social connections (Kindberg et al., 2005; Barasch et al., 2014; Lee et al., 2015; Pittman and Reich, 2016).

Many people share their photos, and if a photo is liked and appreciated by others, the photographer experiences self-efficacy (Krämer and Winter, 2008) and self-esteem (Burrow and Rainone, 2017). This is exploited by the “like buttons,” an enormously popular feature of social media platforms (Kemp, 2021). Sharing photos contributes to a common understanding of the world. Sharing photos also enables people to convey others views that they enjoy, e.g., photos of hilarious events or natural sceneries. Photos of natural scenes (as opposed to human artifacts or urban environments) have a positive influence on the well-being of viewers (Berto, 2005; Valtchanov and Ellard, 2015) as “Biophilia” is a human universal (Appleton, 1975; Ulrich, 1983; Wilson, 1984; Kaplan and Kaplan, 1989). Viewing such photos relaxes, reduces emotional stress, and thereby regenerates depleted cognitive resources.

People often use photos to show others who they are and what role they play in society. Issues of identity, reputation, prestige, or status often play a role in personal photography (Chalfen, 1987; Barasch et al., 2014; RSPH and YHM, 2017). If one person photographs another person, this can be of value only for the photographer, or for both (Milgram, 1976). People can use photos to influence how other people perceive the pictured individuals, objects, or events and thus exert social control (Sharples et al., 2003; Diefenbach and Christoforakos, 2017). People being photographed, however, may also use this circumstance for their own goals, like for influencing how others perceive them (Harrison, 2002; Krämer and Winter, 2008; Jain and Mavani, 2017). Being photographed can immensely increase the size of the “audience.”

As humans are radically social in their nature, observing or monitoring the behavior of other people plays a central role in the motivation to use social media on the internet and to post photos (Joinson, 2008; Lee et al., 2015; Malik et al., 2016). People are usually aware of the presence of cameras. This may produce “audience effects,” i.e., the feeling of being watched influences behavior and makes people behave in a socially agreeable way (Bateson et al., 2006; Oda et al., 2015), by showing, for example, “photo faces.” Through this tendency, photography supports cooperative coexistence in complex societies and has an adaptative value both, on the individual and on the societal level.

We are well aware that people also distribute photos of atrocities. The impact of such photos can be used to boost the importance of the photographer or distributor, or even to hurt other people, to violate the rights of others, or to deceive (Smith et al., 2008; Kowalski et al., 2014). Manipulative to harmful photo use is facilitated by the fact that photographic forgeries are becoming increasingly difficult to detect, both in social and in journalistic media (Campbell, 2014; Nightingale et al., 2017). Various detrimental outcomes of taking and using photos have required legal regulation of photography-related behaviors [e.g., Regulation (EU), 2016]. The ubiquity of taking photos has massively reduced possibilities of intimacy and privacy. A vast dark side of photography exists outside of personal experience. The large social media providers use the shared personal photos as a data source. The acquisition of these data, their possession, the algorithms of their management and the extraction of information from them give the companies enormous power, which has not been put under democratic control until now (Zuboff, 2015).

#### Courtship and Mating

Courtship and mating are certainly part of the domain of establishing and strengthening social connections and attachment (Hazan and Shaver, 1987; Fisher et al., 2002). But they are directly relevant in terms of evolutionary function and as such encompass a range of distinct strategies and conflicts (Fisher et al., 2002; Buss and Duntley, 2011). The global prevalence of intimate partner homicide reflects the high value of the activities and resources that are at stake, as well as the severity of the conflicts in question (Stöckl et al., 2013). Sexual or reproductive behaviors shaped all living beings and played a central role in the evolution of human cognition (Miller, 2001; Nowak, 2006). Sexual themes and symbols are featured in some of the oldest preserved artifacts (Conard, 2009). With photos, a new type of visual cueing was developed that fulfills special functions in attracting potential partners, mate selection, and sexual behavior. The potentials of photography range from tender romantics to hardcore pornography.

“Beauty” plays a special role in this context. Many people want to take and use beautiful photos (Bakhshi et al., 2015; De Looper, 2016). Darwin (1879/2004) associated the “sense of beauty” (p. 114) with the context of sexual selection: the function of beauty is that the choosing female or male individuals are “excited” by it. Individuals considered to be beautiful manage to “excite attention” (p. 467). In this sense, beauty is a sensory signal that it could be advantageous to pay attention to, and approach, the sender of this signal. Among the hashtags (terms assigned to posted photos) that were most frequently used on Instagram in 2020, “Love” came first, “Art” fifth, and “Beautiful” sixth (Kemp, 2021). Instagram is the most photography-related social platform and was the fifth most visited website worldwide in 2020 (Kemp, 2021).

The invention of photography and its further technological developments, including digital communication, allowed people to create a new type of sensory cues relevant to courtship activities, mate selection, sexual intercourse, and (ultimately) reproduction. Photography has been used almost from the start to satisfy cravings for pictures of naked people and for erotic images. Retinal images of naked potential partners expressing interest in sexual activity has meant observers had access to reproduction for hundreds of thousands of years. Photos of sexual acts are among those images that are most emotionally arousing (Bradley and Lang, 2007; Wehrum et al., 2013) and pornography is one of the most prominent domains of internet use.

People also use photos to influence choices of potential romantic or sexual partners. The success of dating applications on the Internet has greatly increased the importance of photos in connection with courtship and mating (Piazza and Bering, 2009; Smith, 2016; Hobbs et al., 2017). Social media platforms and dating apps enable users to form relationships with people they have never seen before. Mobile dating applications are used by more and more people (Smith, 2016; Morris, 2021). People looking for partners create profiles on these apps that they use to present themselves. Photos of oneself play a central role in this. People show photos of themselves—often also taken by themselves—in which they are represented as they would like to be seen by potential romantic partners (Sedgewick et al., 2017; Gale and Lewis, 2020). The use of such photos enables people to reveal actual traits of themselves, but also to make themselves appear more attractive than they potentially are (Sedgewick et al., 2017; Gale and Lewis, 2020). People can also use symbolic self-made photos to create a desirable impression of themselves in potential romantic partners, for example photos of groups of nice, laughing people, pets, flowers, a beautiful garden, an elegant apartment, tourist attractions, dangerous environments, sporting events, or full bookshelves (Krämer and Winter, 2008; Piazza and Bering, 2009). Online dating is not only increasing rapidly among young adults, but also among the older population (Smith, 2016; Morris, 2021). Through dating apps, photos play an increasingly important role in mate selection, which played a central role in the evolution of human cognition (Miller, 2001). When photos are used in dating and courtship, there is also the characteristic connection between emotionally positive information and the processing of uncertainty (Berger and Calabrese, 1975; Knobloch and Solomon, 1999), addressed above. In this context, the positive information concerns one's own attractive properties. Uncertainty is associated with one's search, and potential negative outcomes of establishing relationships with people one does not know from face-to-face encounters.

## Discussion

### The Mental Utilization Hypothesis of Photography

We propose that the success of smartphones as well as photography is based on core human mental mechanisms which are primarily related to the social domain. Photography exploits evolved cognitive and social predispositions. In this sense, our framework is a mental exploitation hypothesis, analogous to the Sensory Exploitation Hypothesis in evolutionary biology (e.g., Ryan, 1990). This hypothesis states that new preferences evolve along established pre-existing sensory biases and response tendencies, such as primates owing their social and/or sexual preference for red to their old predilection for this color, which usually indicates ripe fruits (Ghazanfar and Santos, 2004).

Sensory biases and preferences also play an important role in photography-related behaviors. The visual channel provides information, which is converted into, or affects, mental representations. In our framework, however, the focus is on a higher, more integrated level of processing, on which those mechanisms and functions are organized that control the mental representation of the world and flexibly adapt social behavior. In connection with photography, the term exploitation may have a negative connotation, such as photographers exploiting the people in front of the camera (e.g., Sontag, 1978). For this reason, we refer to our framework as the mental utilization hypothesis of photography. It suggests that photography fits the nature of human perception and mental processing like a key fits its lock.

### Photography as a Coping Strategy

Along to the four levels of Tinbergen, our analysis of photography-related behaviors suggests that people take and use photos to cope with certain stressful and threatening events in specific ways. The conceptualization of photography as a coping strategy is counterintuitive against the background that people usually like to take photos and generally take, share and own photos of events associated with happiness, pleasure, love, or success (Chalfen, 1987; Sharples et al., 2003; Hu et al., 2014). Individuals who take or use such photos, we propose, experience a pleasant situation, but are also—non-consciously—exposed to threatening information or uncertainty. As examples, we mentioned the mother who photographs her 6-year-old son, the lonely hiker, and the man who is afraid of flying. Taking and using photos allows people to search for, and engage, in emotionally positive information. Successful coping through photography-related behaviors reduces complexity, uncertainty, and anxiety. Coping, or the exercise of cognitive control, does not have to be exclusively reactive, but can also be carried out proactively (Bandura, 1997). Coping through taking and using photos has features that can be described on a continuous scale, with reactive coping at one end and proactive coping at the other.

People use photography not only to cope with events with generally positive emotional value, but also in coping with negative events. For example, traffic accidents, high-rise fires or other disasters tend to lure in bystanders and onlookers taking smartphone photos of the scene or of the victims (Vollmuth, 2017; Newton, 2019). There is no research on the motives which drive such photography-related behaviors. They may be similar to the motives which make people watch horror or crime films (Bartsch and Mares, 2014). What people see confronts them with something extremely meaningful—threats that exist in the world, their own mortality and vulnerability (Arndt et al., 1997). Most of these bystanding photographers immediately share their products. Taking and sharing the photos, we suggest, enable people to make sense of threatening events to get along with them, but also use them to push their own importance and prestige within their networks.

### Has Photography Become a Human Universal?

Several researchers discussed the creation and use of representational pictures as human universal (Deacon, 2006; Donald, 2006; Dutton, 2009). The creation of realistic visual pictures appeared more than 30,000-years ago and some of them have been preserved on cave walls (Guthrie, 2005; White et al., 2018). Photography, in connection with digital technology and smartphones, has made it possible for everyone to create, own, and share realistic pictures easily and effortlessly and to integrate such pictures in everyday life. Based on our analysis and statistical data (Statista, 2019; Carrington, 2020; Kemp, 2021), we conclude that taking, viewing, and sharing photos through the use of smartphones has become a human universal—a “new” universal, according to Brown's (1991) classification—that is based on context-sensitive predispositions, particularly connected with the radically social human nature, and differentiates itself in the societal domain (Kotrschal, 2019). Photography not only classifies as a human universal, but also as a unique human feature not shared with any other animal species—not only because other species lack the technical means of photography, but before all, they seem to lack the motivation and mental mechanisms behind the typical human urge to capture the world in images. We conclude that photography is closely matching the unique construction of the human mind and qualifies as a feature of human nature, i.e., the *Conditio humana* (Arendt, 1958; Kotrschal, 2019).

**Figure 3:**
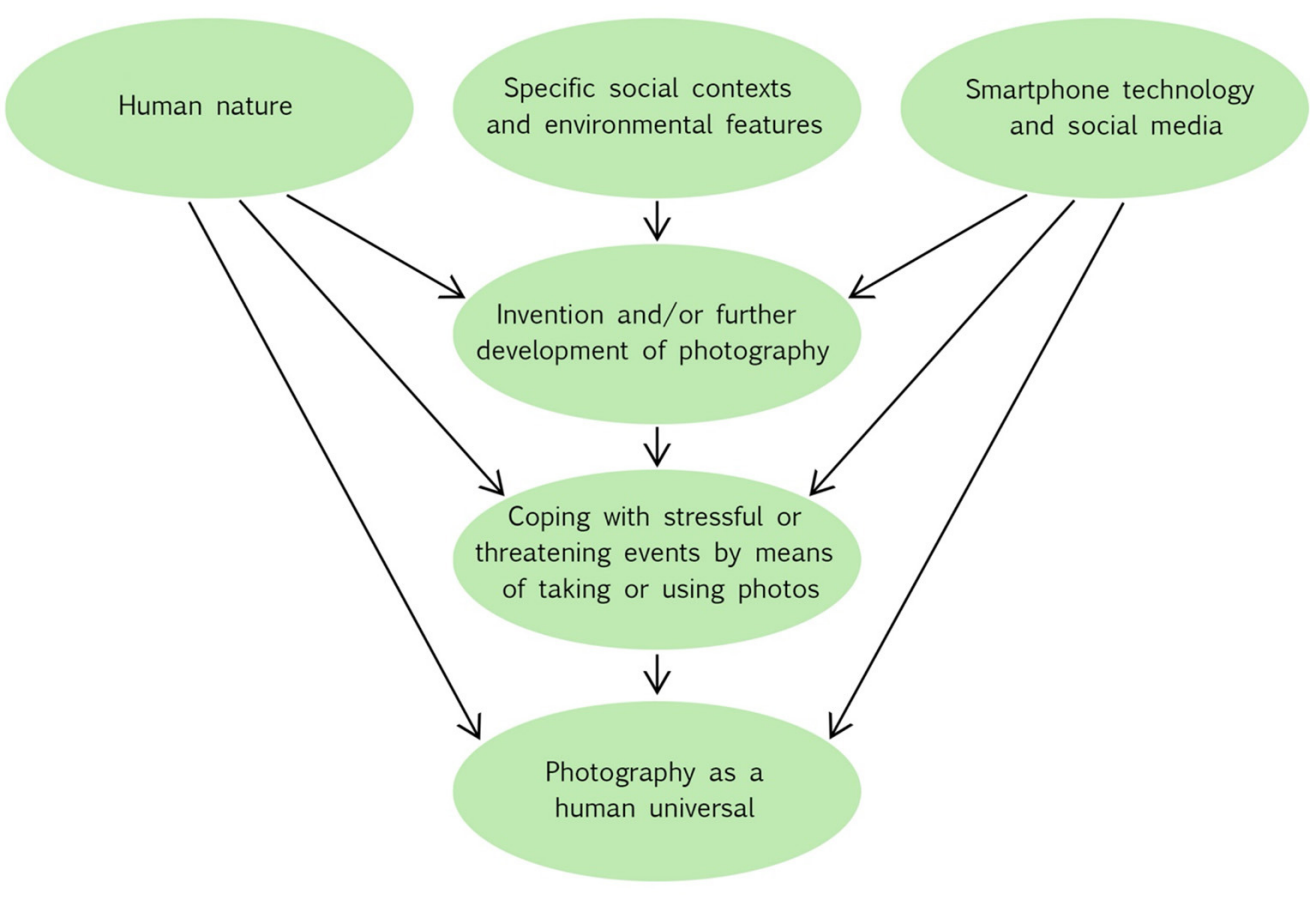
The Mental Utilization Hypothesis of Photography. The schematic illustration shows the proposed conditions, components, and abilities that made photography a human universal. An arrow means “provides the basis for” or “leads to”.

Figure 3 summarizes the conditions, components, and abilities that have made photography a human universal as proposed by the mental utilization hypothesis of photography. One element of Figure 3 relates to the specific social contexts and environmental features that generate photography-related behaviors, as suggested by the evolutionary building blocks of photography. They are (1) coexistence in large, complexly structured societies; (2) frequent encounters with strangers, the outcome of which is often difficult to predict; (3) strong mutual observation of behavior; (4) individuals' well-being and prosperity depend on judgments by strangers; (5) requirement to display one's own status symbolically in public; (6) continuous confrontation with the news of success or profit, as well as disaster, illness, or death; (7) large number of potential sexual or reproductive partners among strangers; (8) individuals have to make far-reaching decisions about their future lives; (9) requirement of communication with absent or distant people; and (10) requirement of quick communication with strangers across cultural or linguistic boundaries.

### Limitations

The analysis of a particular behavior on the basis of the four levels of Tinbergen requires the integration of findings from a range of disciplines. Despite the referenced mechanisms and functions of taking photos, which represent the present state of knowledge, our conclusions remain necessarily speculative—because of the preliminary nature of all scientific results, because of the inherent pitfalls of attempting to integrate such diverse results into a comprehensive synthesis, and due to the space constraints of a journal article. In addition, there are very few empirical findings on taking photos, and they come only from the Western world. Thus, we may underestimate the cultural diversity in photography, although we are quite confident that the behavioral core is based on human nature, and therefore, should in principle, apply to all people. Within our conceptual frame we describe taking and using photos as functional outcomes of cognitive and social adaptations. It could certainly be argued that the success of photography is ultimately a byproduct of the accessibility, affordability and success of smartphones and social media, which results from marketing activities of powerful companies. But this is a different level argument not contradicting our utilization hypothesis. Our analysis of photography-related behaviors as coping strategies creates a picture of photography in which the benefits are generally greater than the cognitive and social costs, which also explains why photography became such a sweeping worldwide success once the smartphone technology became available.

The goal of producing an image that supports memory only plays a subordinate role in our description of photography-related behaviors. In this respect, our framework differs from explanations that describe the production of memory pictures as a central function of photography (Milgram, 1976; Kahneman, 2011; Frohlich et al., 2013; Henkel, 2014). These explanations are consistent with the fact that many people stated the retention of memories, when asked about the purpose of photographing (Chalfen, 1987; Kindberg et al., 2005; Broekhuijsen et al., 2017; Lee, 2018). Empirical findings, however, show that people lose many photos they have taken or never look at them again (Kirk et al., 2006; Whittaker et al., 2010). Furthermore, the experimental studies on the question of whether taking or seeing photos improves people's ability to remember past events produced a multitude of different and sometimes contradicting results (for review, see Foley, 2020). This was one of the incentives for us to attempt a new synthesis within an evolutionary theory frame.

### Testable Predictions for Future Research

As shown in Table 2, the mental utilization theory of photography allows generating a number of testable predictions. Ideally, these would be tackled by experimental and behavioral field studies in natural environments, in both everyday and lab situations. Rapidly developing mobile techniques (such as EEG headsets, eye-tracking devices, etc.) open up new possibilities for the investigation of the attention structures and specific cognitive mechanisms involved in taking and using photos.

**Table 2:** A sample of testable predictions along the 4 levels of Tinbergen based on the mental utilization hypothesis of photography.

1. Making sense
1a. Mechanisms: Studies of the neural substrates of taking photos will find that photographers make early basic decisions quickly and non-consciously. Researchers will also observe that photography-related behaviors and brain activity will utilize mechanisms that integrate external, emotionally positive information and internal, threatening information. The neural substrates of cognitive control and the regulation of emotions play a crucial role in this
1b. Ontogeny: Children will preferentially be interested in photos of environments, behaviors, and events that they will soon face and that have both positive and threatening traits, and will prefer social contexts with humans and animals. Parents will take photos of their children especially in contexts where there are both positive and negative predictions. Older people will prefer photos in which their decisions and lives appear meaningful and successful
1c. Evolutionary history: Despite increasing knowledge of fake photos, people will tend to believe that pictured events really did take place, as long as the events make sense in relation to their desires and experiences
1d. Functions: People will prefer to take and use personal photos in situations in which they perceive emotionally positive events that they also associate with stress or threat. That way, taking photos will help people cope with social stress. If people want to convince others of the special importance of an object or event they will use photos more often than video clips
2. Appropriating an image
2a. Mechanisms: Neuroscientific studies will find that photographing involves activities of brain structures that form the neural basis of appropriation and possession
2b. Ontogeny: People will prefer photography-related behaviors when they are non-consciously processing the appropriation of a resource. In connection with identity, the importance of owning personal photos increases with age
2c. Evolution: Collecting personal photos of events that the owners associate with beauty and/or success will enhance the owners' well-being
2d. Functions: People will prefer ownership of self-made personal photos to photos taken by others of the same object or event, even if their own photos are of inferior quality
3. Establishing and supporting social connectedness
3a. Mechanisms: Neurobiological studies will find that taking and using personal photos involves nodes and activities in the social behavior network in the brain—not only “wanting” and “liking” responses, but also mechanisms that are related to the processing of representations of being connected, alone, isolated, or abandoned
3b. Ontogeny: Children will prefer to take and use photos of events that are relevant to their natural and social environment, especially family. Young people and adults will prefer to take, possess and use photos that show animals and people with whom they are, or want to be, emotionally connected. Older people will surround themselves in their home with photos of people who are or were important to them
3c. Evolution: Viewing photos of close relatives and friends will have a positive effect on the well-being of the viewers at times when the pictured people are absent. This will entail a supportive effect on societal and biological fitness. Photos that evoke associations of pictured individuals or groups with social attachment, supportive relationships, and cooperation in viewers, will support the success of the pictured individuals or groups in societies. Variations in photography-related behaviors will change the environments in which they are performed, for example, as the increasing presence of cameras in public spaces influences people's behavior
3d. Functions: Seeing photos depicting people with whom the viewers are connected by a close emotional relationship will strengthen the sense of social connectedness and provide a sense of security and calmness. Photos of oneself will be more efficient than verbal descriptions or video clips when the goal is influencing or controlling the characteristics that other people associate with oneself
4. Courtship and mating
4a. Mechanisms: In the brains of people who are looking for sexual or romantic partners, seeing photos of potential partners will elicit intense motivational reactions, which are related to partner attraction and sexual arousal. There will be quantitative gender differences in this
4b. Ontogeny: Sexually mature individuals of all ages will want to appear attractive in photos
4c. Evolution: A stock of personal photos associated with beauty and success that a person owns will be recognized by potential partners as a valuable resource and directly or indirectly support the reproductive success of the owner
4d. Functions: In a mating competition in social environments, in which information is exchanged without direct personal encounters, people who use photos to represent themselves as mates will be more successful than people who use words or video clips

## Conclusion

We position viewing, sharing, and using personal photos within the coherent theory of the evolution of life and human nature. On the basis of the four levels of Tinbergen (1963), we developed a theoretical framework that describes the characteristics of photos and photography-related behaviors, including potential adaptative values related to the evolutionary functional domains of coping, well-being, social connectedness, courtship, and mating. We hypothesized that people take or use photos in contexts in which a pleasant event is coupled with uncertainty or with the processing of threatening information, and that people generally use photography as a coping strategy. Based on our analysis, we propose the Mental Utilization Hypothesis that explains the success of photography by its match with core human mental mechanisms, which characterize human nature.

The proposed hypothesis provides a novel conceptual framework, potentially useful in devising future experimental studies of photography. Despite the global ubiquity of photos, there is still almost no research into the cognitive mechanisms underlying photo taking. Investigations into the courtship or mating functions of photography are still limited to the explicit use of photos in online dating, but these functions are more fundamental and embracing. Studies regarding evolutionary functions of photography are particularly desirable. Important findings could be gained through comparisons between cultures, subcultures and sociological strata, gender and age classes. Important questions in such comparisons could be whether social prestige and social, occupational, or reproductive success can be linked with photography. Is photography an addition to existing social and sexual behavior or is it part of a socio-sexual change which compensates for or replaces previous behaviors or customs? Does it have “emergent properties” not found in its constituent elements? Last but not least, our description of taking and using photos as a coping strategy provides a comprehensive theoretical basis for new experimental research into the application of photography in psychotherapeutic contexts. With photography, people developed a new means of representing experiences and ideas through pictures with special characteristics, the understanding of which requires a minimum of effort and cultural knowledge. We are creatures in an increasingly complex social world for whom and in which these pictures open up powerful possibilities for action, but also for feeling at home and safe.

## Data Availability Statement

The original contributions presented in the analysis are included in the article, further inquiries can be directed to the corresponding author.

## Author Contributions

All authors listed have made a substantial, direct and intellectual contribution to the work, and approved it for publication.

## Conflict of Interest

The authors declare that the research was conducted in the absence of any commercial or financial relationships that could be construed as a potential conflict of interest.
